# Effects of Waterlogging on Leaf Mesophyll Cell Ultrastructure and Photosynthetic Characteristics of Summer Maize

**DOI:** 10.1371/journal.pone.0161424

**Published:** 2016-09-01

**Authors:** Baizhao Ren, Jiwang Zhang, Shuting Dong, Peng Liu, Bin Zhao

**Affiliations:** State Key Laboratory of Crop Biology and College of Agronomy, Shandong Agricultural University, Taian, Shandong, PR China; University of Tasmania, AUSTRALIA

## Abstract

A field experiment was performed to study the effects of waterlogging on the leaf mesophyll cell ultrastructure, chlorophyll content, gas exchange parameters, chlorophyll fluorescence, and malondialdehyde (MDA) content of summer maize (*Zea mays* L.) hybrids Denghai605 (DH605) and Zhengdan958 (ZD958). The waterlogging treatments were implemented for different durations (3 and 6 days) at the third leaf stage (V3), the sixth leaf stage (V6), and the 10^th^ day after the tasseling stage (10VT). Leaf area index (LAI), chlorophyll content, photosynthetic rate (*P*_n_), and actual photochemical efficiency (*Φ*_PSII_) were reduced after waterlogging, indicating that waterlogging significantly decreased photosynthetic capacity. The chloroplast shapes changed from long and oval to elliptical or circular after waterlogging. In addition, the internal structures of chloroplasts were degenerated after waterlogging. After waterlogging for 6 d at V3, the number of grana and grana lamellae of the third expanded leaf in DH605 were decreased by 26.83% and 55.95%, respectively, compared to the control (CK). Those in ZD958 were reduced by 30.08% and 31.94%, respectively. Waterlogging increased MDA content in both hybrids, suggesting an impact of waterlogging on membrane integrity and thus membrane deterioration. Waterlogging also damaged the biological membrane structure and mitochondria. Our results indicated that the physiological reactions to waterlogging were closely related to lower LAI, chlorophyll content, and *P*_n_ and to the destruction of chloroplast ultrastructure. These negative effects resulted in the decrease of grain yield in response to waterlogging. Summer maize was the most susceptible to damage when waterlogging occurred at V3, followed by V6 and 10VT, with damage increasing in the wake of waterlogging duration increasing.

## Introduction

Waterlogging is a major source of abiotic stress in crop production. Globally, it is estimated that 10% of all irrigated land is affected by waterlogging, which might reduce crop productivity as much as 20% [1]. The disaster zones within the Yangtze Watershed and the Huanghuaihai Plain represent approximately 75% of the total disaster area in China [2]. In the Huanghuaihai Plain, most rainfall occurs during the growing season of summer maize, and the growth and yield of summer maize are significantly affected by excessive rainfall and/or flooding [3].

Excessive soil moisture leads to poor soil aeration, which not only limits root growth, reduces leaf emergence rate, and disorders root growth [4], but also leads to the destruction of root physiological function, thus resulting in alteration of plant hormone balance and nutrients shortage [4, 5]. Waterlogging also enhances anaerobic respiration, leading to the accumulation of a large number of harmful substances (e.g., H_2_S, FeS) in the soil. The rhizosphere environment deteriorates, resulting in the reduction of mineral ions and beneficial trace element absorption, ultimately reducing root growth and development [5]. Waterlogging significantly decreases the activity of superoxide dismutase (SOD), peroxidase (POX), and catalase (CAT), damaging the protective enzyme system, and it increases malondialdehyde (MDA) content, suggesting an impact of waterlogging on membrane lipid peroxidation and integrity and thus membrane deterioration, accelerating leaf senescence [6, 7]. Waterlogging also decreases soluble protein content, thus influencing carbon assimilation, and it degrades chlorophyll, resulting in the decline of photoassimilation [8]. Under waterlogging conditions, maize leaves have to suffer stomatal closure, reductions in transpiration and photosynthetic rates, and leaf blade wilting. With the extension of waterlogging duration, chlorophyll content, the related photosynthetic enzymes [7], and PSII photochemical efficiency were reduced [9], resulting in a significant yield reduction [3].

Currently, most previous studies have focused on effects of waterlogging on grain yield and plant growth of summer maize [3, 8]. However, few studies have reported effects from different durations of waterlogging at various stages on leaf photosynthesis characteristics at the cellular level. Under adverse circumstances, chlorophyll content and photosynthetic capacity are significantly reduced, mainly due to damages on chloroplast morphology and ultrastructure of functional leaves [10, 11]. The morphology and internal structure of mesophyll cells, a fundamental component of photosynthesis, play an important role in photosynthetic capacity. Chloroplasts [12] and mitochondria [13] of all organelles in mesophyll cells are the most sensitive to light quantity, and their morphology and internal structure change in response to environmental variation [14]. Therefore it is important to investigate waterlogging effects on leaf photosynthesis characteristics at the cellular level. In this paper, our objective was to explore the effect of waterlogging for 3 or 6 days on leaf mesophyll cell ultrastructure and photosynthetic characteristics of summer maize at different growth stages. The effects of waterlogging for 3 or 6 days at the third leaf stage (V3), the sixth leaf stage (V6), and the 10^th^ day after the tasseling stage (10VT) on mesophyll cell ultrastructure, chlorophyll content, leaf gas exchange parameters, fluorescent characteristic, MDA content, and grain yield were observed.

## Materials and Methods

### Plant materials and experimental location

Summer maize hybrids Denghai605 (DH605) and Zhengdan958 (ZD958) were used for experimental materials, which are planted most popularly in China. The field experiment was conducted in 2013 and 2014 at the State Key Laboratory of Crop Biology and the experimental farm of Shandong Agricultural University, China (36°10’N, 117°04’E, 151 m a.s.l.). There is a temperate continental monsoon climate in the experimental region. The effective accumulated temperature of summer maize growth periods in 2013 and 2014 was 1673.1°C d and 1741.0°C d, respectively. The mean total precipitation that occurred during summer maize growth periods in 2013 and 2014 was 401.3 mm and 356.0 mm, respectively. The experimental soil type is brown loam. Concentrations of organic matter, total N, rapidly available phosphorous (P), and rapidly available potassium (K) in the upper 20 cm of soil were 10.71 ± 0.79 g kg^-1^, 0.89 ± 0.19 g kg^-1^, 50.65 ± 1.27 mg kg^-1^, and 86.15 ± 1.13 mg kg^-1^, respectively.

### Experimental design

Each plot was 4 m × 4 m and separated by polyvinyl chloride (PVC) boards of 4 m×2.3 m as water barriers. Every PVC board was buried 2.0 m below the surface and the remaining 0.3 m was above the ground. Maize was sown on June 16 for both years with a plant density of 67,500 plants ha^-1^. There was a water pipe to supply water in each waterlogged main pool. The water in waterlogged pool was maintained at 2–3 cm above soil surface through water valve to control water flow during waterlogging period. Experimental treatments contained of different waterlogging stage (the third leaf stage (V3), the sixth leaf stage (V6), and the 10^th^ day after the tasseling stage (10VT)), waterlogging duration (3 d and 6 d), and no waterlogging (CK). In the CK, soil moisture was kept optimum during the whole growth period. Details of the experimental treatments are shown in Table 1. Each treatment was replicated 3 times in a completely randomized block design. Disease, weeds, and pests were well controlled in each treatment. The herbicide of 90% atrazine acetochlor was diluted 2000–3000 times and sprayed on the whole field surface by 600 L ha^-1^ before germinating to control weeds; the pesticides of 50% phoxim emulsifiable concentrate were diluted 1000 times by water and sprayed by 750 L ha^-1^ at the ninth leaf stage (V9) to control corn borers.

**Table 1:** Waterlogging treatments in the field from 2013 to 2014.

Hybrid	Code	Treatment
**DH605**	V3-3	Waterlogging for 3 days at the third leaf stage
**ZD958**	V3-6	Waterlogging for 6 days at the third leaf stage
	V6-3	Waterlogging for 3 days at the sixth leaf stage
	V6-6	Waterlogging for 3 days at the sixth leaf stage
	10VT-3	Waterlogging for 3 days at the 10th day after the tasseling stage
	10VT-6	Waterlogging for 6 days at the 10th day after the tasseling stage
	CK	No waterlogging

### Leaf area index

Fifteen representative plant samples were marked from each plot at V6, the twelfth leaf stage (V12), tasseling stage (VT), milk stage (R3), and physiological maturity stage (R6) to measure leaf length (*L*) and maximum leaf width (*W*) for the largest leaf on the individual tagged plants, and then leaf area and leaf area index (LAI) were calculated according to the method of Montgomery [15].

Leaf area=L×W×0.75

LAI=(leaf area per plant×plant number per plot)/plot area

### Chlorophyll content and MDA content

The latest fully expanded leaves of three plants were sampled after waterlogging at V3 and V6, ear leaf was sampled after waterlogging at 10VT. Washed fresh leaves (± 0.50 g) were homogenized in 5 ml of 50 mmol L^-1^ potassium phosphate buffer (pH 7.8). The homogenate was filtered through muslin cloth and centrifuged at 15 000 × g for 20 min at 4°C, and the supernatant was immediately used for the malondialdehyde (MDA) content.

The absorbance of supernatant was monitored at 532 and 600 nm using ultraviolet spectrophotometer (UV-2450, SHIMADZU, Japan). After subtracting the non-specific absorbance (600 nm), the MDA concentrations were calculated by means of an extinction coefficient of 156 mmol L^-1^ cm^-1^ and the formula [16]:

$$\text{MDA }(\mu \text{mol MDA }{\text{g}}^{-1}\text{FW})\;=\;[(A_{\;532}-A_{600})/156]\;\times 103\;\times \text{ dilution}.$$

The chlorophyll a and b contents were assessed using a spectrophotometer at 663 and 645 nm, respectively, after the leaves had become white due to soaking in 15 ml of 95% ethanol for 48 h. The soaking was made in the darkness. The chlorophyll contents were calculated according to Li’s method [17].

$$\text{Chl}\;\text{a}=12.72A_{663}-2.59A_{645}$$ (1)

$$\text{Chl}\;\text{b}=22.88A_{645}-4.67A_{663}$$ (2)

Chl (a+b)=Chl a+Chl b (3)

### Leaf gas exchange parameters and chlorophyll fluorescence parameters

At the next day after the end of waterlogging treatments, the photosynthetic rate (*P*_n_), transpiration rate (*T*_r_), stomatal conductance (*G*_s_) and intercellular CO_2_ concentration (*C*_i_) of the functional leaf were measured using a portable infrared gas analyzer (CIRAS II, PP System, Hansatech, UK). Measurement conditions were kept consistent: LED light source, and the PAR was 1 600 μmol m^-2^. CO_2_ concentration was maintained at a constant level of 360 μmol mol^-1^ using a CO_2_ injector with a high-pressure liquid CO_2_ cartridge source.

Chlorophyll fluorescence was measured with a FMS-II pulse modulated fluorometer (Hansatech, UK) on the same leaves as used for gas exchange measurements. Minimal fluorescence (*F*_0_) was measured under a weak pulse of modulating light over a 0.8 s period, and maximal fluorescence (*F*_m_) was induced by a saturating pulse of light (8000 μmol m^-2^ s^-1^) applied over 0.8 s. The maximal quantum efficiency of PSII was determined as *F*_v_/*F*_m_, where *F*_v_ is the difference between *F*_0_ and *F*_m_. An actinic light source (600 μmol m^-2^ s^-1^) was then applied to achieve steady-state photosynthesis and to obtain *F*_s_ (steady-state fluorescence yield), after which a second saturation pulse was applied for 0.7 s to obtain *F*’_m_ (light-adapted maximum fluorescence). Fluorescence parameters were calculated by the FMS-II, based on the dark-adapted and light adapted fluorescence measurements. The quantum efficiency of PSII (*Φ*_PSII_) was calculated as (*F*’_m_-*F*_s_)/*F*’_m_ [18].

Five plants per treatment were randomly selected for measurement of photosynthesis and fluorescence parameter from 10:00 AM to 12:00 PM.

### Transmission electron microscope (TEM) sample preparation and observation

The functional leaves from five plant samples were obtained from the center of each plot at the next day after the end of waterlogging treatments. Square leaves (0.5 cm×0.5 cm) were taken near the center vein of each leaf. After fixation with 2.5% glutaraldehyde for 4 h, leaf cells were post-fixed with osmic acid at 4°C for 4 h and then dehydrated with ethanol. When embedded in spurr resin at 70°C for 8 h, thin sections were cut from leaf samples with an LKB-V ultramicrotome and were placed upon 250-mesh grids. Samples were double stained using stem uranyl acetate and lead citrate, and then observed and randomly photographed using a Hitachi-600 transmission electron microscope [13].

### Grain

At R6, 30 ears harvested from three rows at the center of each plot were used to determine yield (moisture content was 14%) and ear traits including length, width, weight, row number, kernels per row, bald tip length, cob weight, and cob width.

$$\begin{array}{l} \text{Grain}\;\text{yield}\;\left({\text{kg}\;\text{ha}}^{-1}\right)\\ =\text{Harvest}\;\text{ear}\;\left({\text{ears}\;\text{ha}}^{-1}\right)\times \text{kernel}\;\text{number}\;\text{per}\;\text{ear}\\ \times 1000\;\text{grains}\;\text{weight}\;\left(\text{g}\;1000{\text{grains}}^{-1}\right)/10^{6}\times (1-\text{moisture}\;\text{comtent}\;\text{\%})/\left(1-14\text{\%}\right) \end{array}$$

### Statistical analysis

Analysis of variance (ANOVA) was performed according to the general linear model procedure of SPSS (Ver. 17.0, SPSS, Chicago, IL, USA). Bivariate correlation analysis was also performed. Results are presented as means over 2 years, as the data were consistent over years. The least significant difference (LSD) between the means was estimated at the 95% confidence level. Unless otherwise indicated, significant differences are at P≤0.05. LSD was used to compare adjacent means arranged in order of magnitude. Calculations and linear regressions were performed using a SigmaPlot 10.0 program.

## Results

### Grain yield

Yield significantly decreased with increasing waterlogging duration at different growth stages. Summer maize was most susceptible to damage at V3, followed by V6 and 10VT, with the most significant reduction for waterlogging 6 d at V3, with yield reductions of 30.30% and 30.39% for DH605 and ZD958, respectively, compared with CK. In addition, waterlogging significantly affected yield components. Grains per ear and 1000-grain weight of DH605 were reduced after waterlogging compared to those of CK, with the most significant reduction of 22.22% and 9.51% in V3-6, respectively. ZD958 decreased by 20.40% and 10.83%, respectively (Table 2).

**Table 2:** Effects of waterlogging on grain yield and yield components of summer maize.

Hybrid	Treatment	Ear number	Grains per ear	1000-grain weight	Yield
		(ears ha^-1^)		(g)	(kg ha^-1^)
DH605	V3-3	65,320a	499c	331c	10,756d
	V3-6	64,914c	437d	317d	8,973e
	V6-3	65,278ab	518bc	346b	11,634c
	V6-6	65,132b	495c	338c	10,830d
	10VT-3	65,049bc	567a	364a	13,371b
	10VT-6	65,437a	522b	351b	11,971c
	CK	65,320a	586a	367a	14,016a
ZD958	V3-3	65,043b	519c	309de	10,418d
	V3-6	63,730c	445e	295e	8,380f
	V6-3	65,328a	522bc	324c	11,232c
	V6-6	63,256c	476d	314d	9,490e
	10VT-3	65,375a	543ab	345a	12,240b
	10VT-6	65,016b	527b	333b	11,415c
	CK	65,954a	563a	350a	12,978a
ANOVA					
Waterlogging period (P)		NS	*	*	**
Waterlogging duration (D)		*	**	*	**
P×D		NS	*	*	**

V3-3: waterlogging for 3 d at the third leaf stage; V3-6: waterlogging for 6 d at the third leaf stage; V6-3: waterlogging for 3 d at the sixth leaf stage; V6-6: waterlogging for 6 d at the sixth leaf stage; 10VT-3: waterlogging for 3 d at the 10^th^ day after tasseling stage; 10VT-6: waterlogging for 6 d at the 10^th^ day after tasseling stage; CK: no waterlogging

Values fallowed by a different small letter within a column are significantly different (P = 0.05) according to L. S. D. (t).

NS: Not significant.

* Significant at the 0.05 probability level

** Significant at the 0.01 probability level

### LAI, chlorophyll content, and MDA content

**Fig 1:**
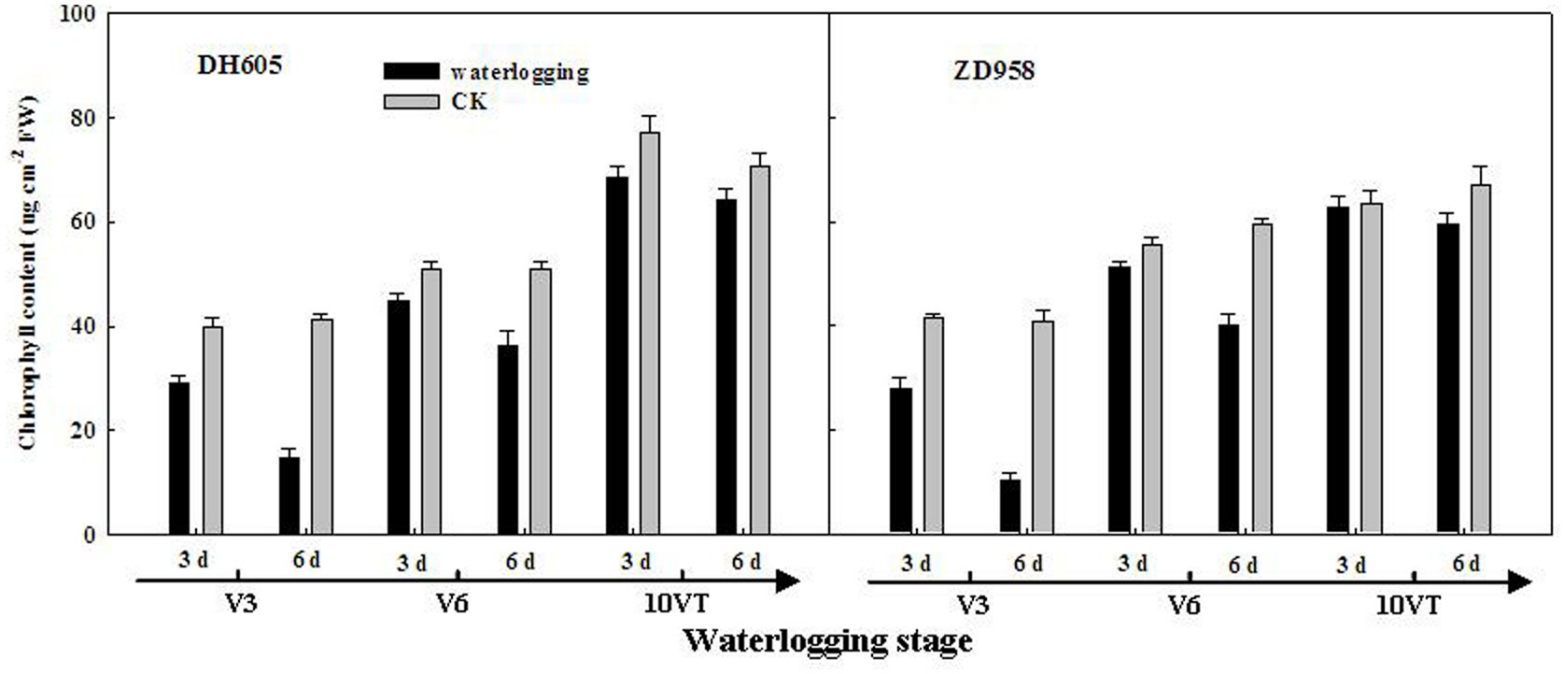
Effect of waterlogging on leaf chlorophyll content of summer maize. V3: the third leaf stage, V6: the sixth leaf stage, 10VT: the 10th day after the tasseling stage.

**Fig 2:**
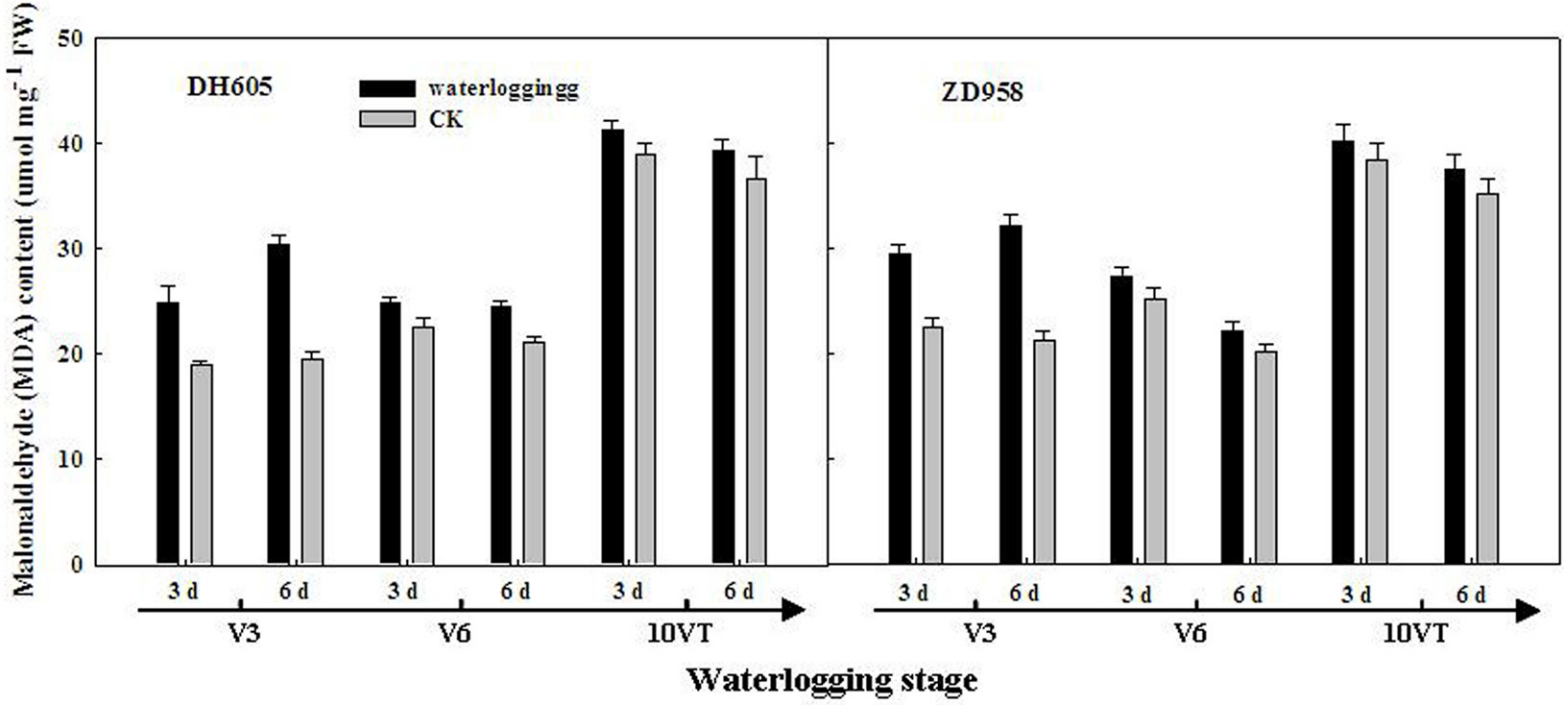
Effect of waterlogging on leaf malondialdehyde (MDA) content of summer maize. V3: the third leaf stage, V6: the sixth leaf stage, 10VT: the 10th day after the tasseling stage.

Leaf area index (LAI) of summer maize was significantly decreased after waterlogging. The negative effects varied with growth stage and duration of waterlogging. LAI was most susceptible to damage when waterlogging occurred at V3, followed by V6 and 10VT. Damage increased with increasing waterlogging duration. LAI of DH605 and ZD958 were decreased by 56.90% and 54.14%, respectively, after waterlogging for 6 d at V3 (Table 3). Moreover, the leaf chlorophyll content of summer maize was significantly declined after waterlogging, following a trend similar to that of LAI. The most significant reduction was found at V3-6, with decreases of 64.30% and 72.36% in the chlorophyll (a+b) content of DH605 and ZD958, respectively, compared with CK (Fig 1). However, MDA content was significantly increased after waterlogging. The negative effects varied with growth stage and duration of waterlogging, and the most significant effect was observed at V3, followed by V6 and 10VT. MDA content increased significantly with increasing waterlogging duration; the most significant change was found at V3-6, with increases of 35.34% and 34.12% for DH605 and ZD958, respectively, compared with CK (Fig 2).

**Table 3:** Effect of waterlogging on leaf area index (LAI) of summer maize.

Waterlogging stage	Waterlogging duration	Treatment	Hybrids	
	(d)		DH605	ZD958
**V3**	3	T	0.14b	0.09b
		CK	0.21a	0.15a
		±CK%	-33.3	-40
	6	T	0.15b	0.13b
		CK	0.35a	0.29a
		±CK%	-57.1	-55.2
**V6**	3	T	1.01b	0.99b
		CK	1.61a	1.38a
		±CK%	-37.3	-28.3
	6	T	1.66b	1.08b
		CK	2.48a	2.03a
		±CK%	-33.1	-46.8
**10VT**	3	T	4.28b	4.34b
		CK	4.36a	4.47a
		±CK%	-1.8	-2.9
	6	T	4.11b	4.28b
		CK	4.36a	4.57a
		±CK%	-5.7	-6.3
**ANOVA**				
**Waterlogging period (P)**			**	
**Waterlogging duration (D)**			**	
**P×D**			*	

T: waterlogging; CK: no waterlogging; ±CK% = (CK value-T value)/CK value×100.

Values fallowed by a different small letter within a column are significantly different (P = 0.05) according to L. S. D. (t).

NS: Not significant.

* Significant at the 0.05 probability level

** Significant at the 0.01 probability level

### Leaf gas exchange parameters

**Fig 3:**
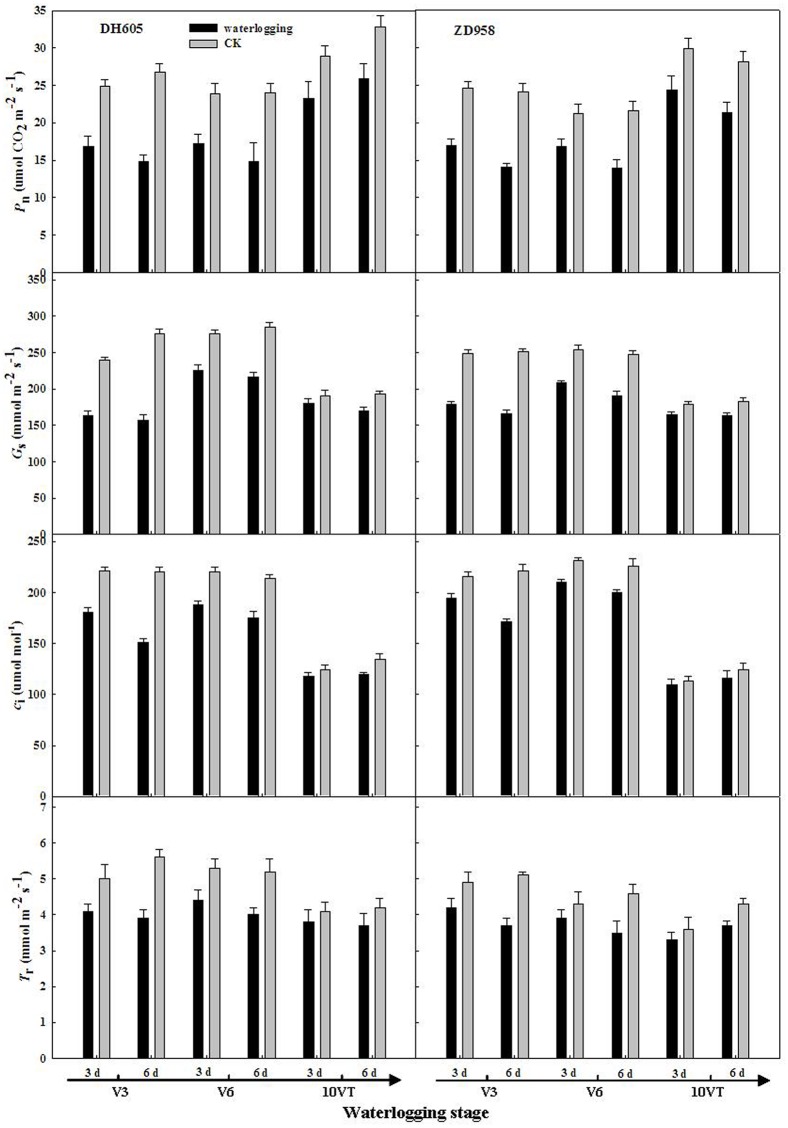
Effect of waterlogging on photosynthetic rate (P_n_), transpiration rate (*T*_r_), stomatal conductance (*G*_s_) and intercellular CO_2_ concentration (*C*_i_) in functional leaves of summer maize. V3: the third leaf stage, V6: the sixth leaf stage, 10VT: the 10^th^ day after the tasseling stage.

Leaf gas exchange parameters of summer maize were significantly affected by waterlogging. The negative effects varied with growth stage and duration of waterlogging, with the most significant effects observed at V3, followed by V6 and 10VT. With increasing durations of waterlogging, *P*_n_, *G*_s_, *C*_i_, and *T*_r_ in treatment V3-6 of DH605 were decreased by 44.68%, 42.97%, 31.4%, and 26.02%, respectively, compared with CK. ZD958 decreased by 41.43%, 33.93%, 22.3%, and 28.43% compared with CK, respectively (Fig 3).

### Chlorophyll fluorescence parameters

**Fig 4:**
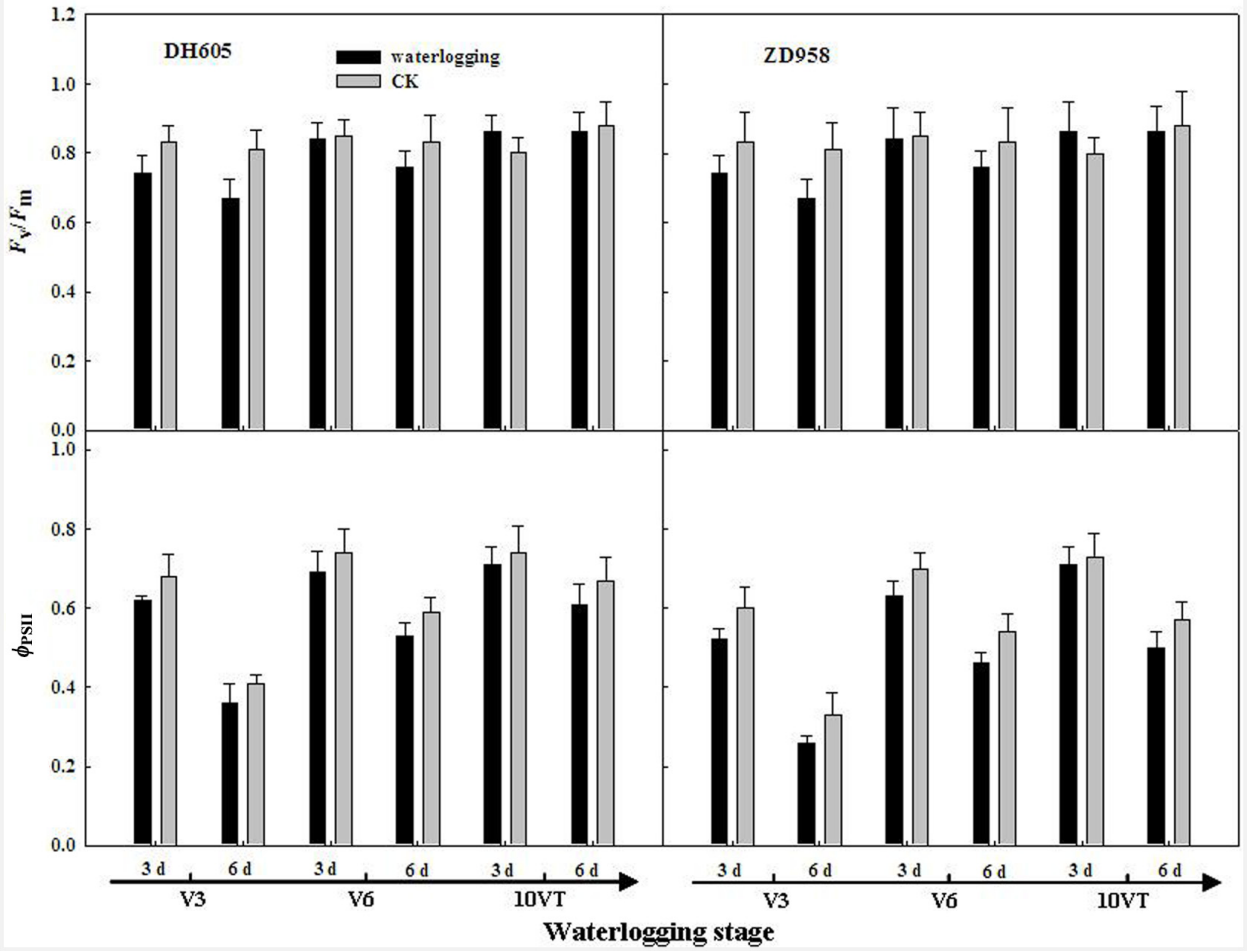
Effect of waterlogging on chlorophyll fluorescence in leaves of summer maize. V3: the third leaf stage, V6: the sixth leaf stage, 10VT: the 10^th^ day after the tasseling stage.

The negative effects of waterlogging on leaf chlorophyll fluorescence parameters of summer maize varied with growth stage and waterlogging duration, the most significant effects were observed at V3, followed by V6 and 10VT. *F*_v_/*F*_m_ and *Φ*_PSII_ were significantly decreased with increasing waterlogging duration, with the most significant reductions in V3-6, with decreases of 16.86% and 11.67% for DH605, respectively. ZD958 decreased by 13.58% and 22.52% compared to those of CK, respectively. The effects of waterlogging for 3 d at 10VT on leaf chlorophyll fluorescence parameters of summer maize were not significant (Fig 4).

### Chloroplast form and its configuration

**Fig 5:**
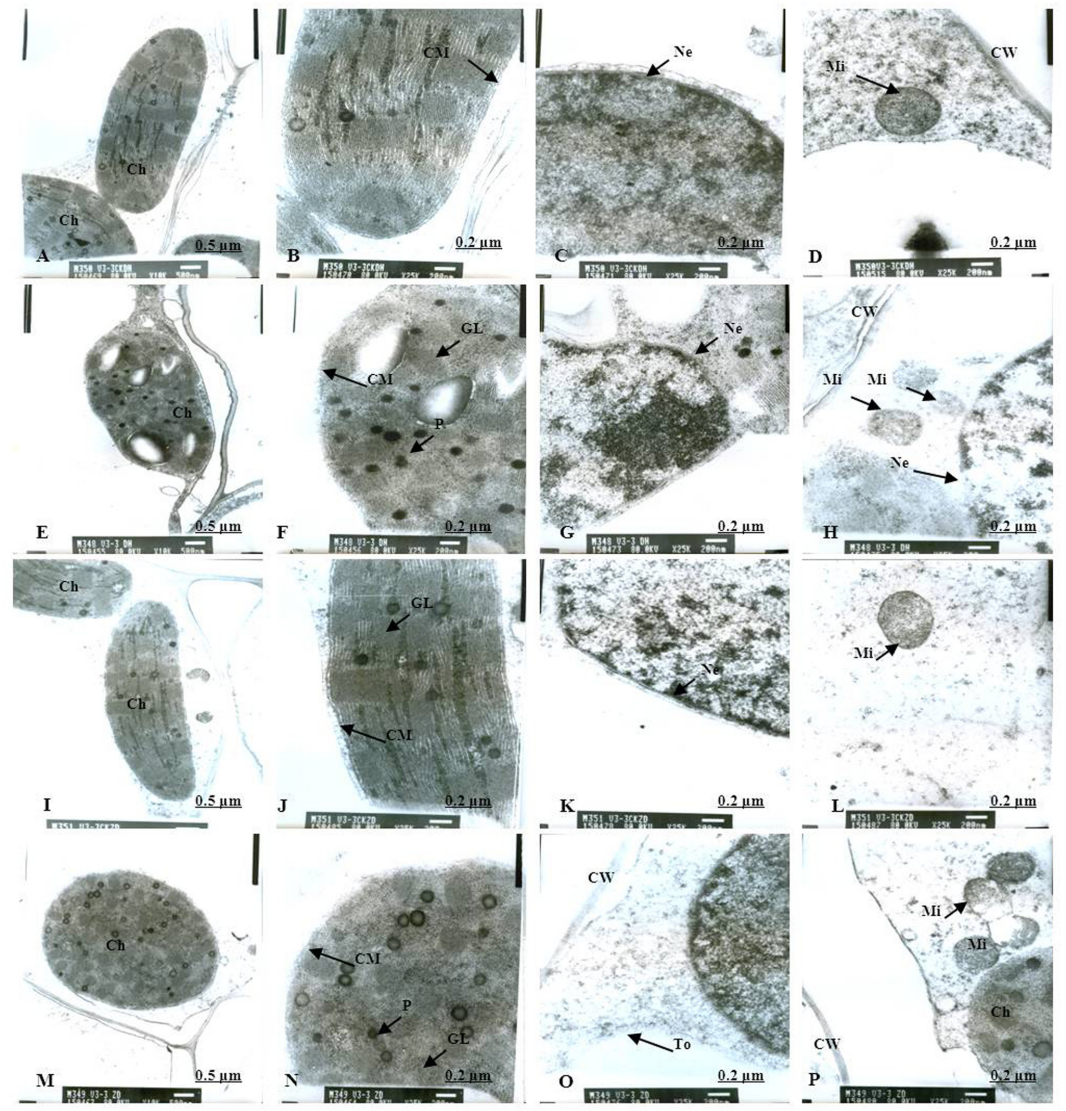
Effects of waterlogging at V3 for 3 d on the mesophyll cell and chloroplast ultrastructure. (A) represents the complete picture of mesophyll cells and topography (×10K) of mesophyll cells in chloroplast of DH605 in CK; (B), (C) and (D) represents ultrastructure (×25K) of mesophyll cells of DH605 in CK; (E) represents the complete picture of mesophyll cells and topography (×10K) of mesophyll cells in chloroplast of DH605 in V3-3; (F), (G) and (H) represents ultrastructure (×25K) of mesophyll cells of DH605 in V3-3; (I) represents the complete picture of mesophyll cells and topography (×10K) of mesophyll cells in chloroplast of ZD958 in CK; (J), (K) and (L) represents ultrastructure (×25K) of mesophyll cells of ZD958 in CK; (M) represents the complete picture of mesophyll cells and topography (×10K) of mesophyll cells in chloroplast of ZD958 in V3-3; (N), (O) and (P) represents ultrastructure (×25K) of mesophyll cells of ZD958 in V3-3; Ch: chloroplast; CM: chloroplast membrane; GL: grana lamella; Mi: mitochondria; Ne: nuclear envelope; P: particles; CW: cell wall. To: tonoplast; MB: multivesicular body.

**Fig 6:**
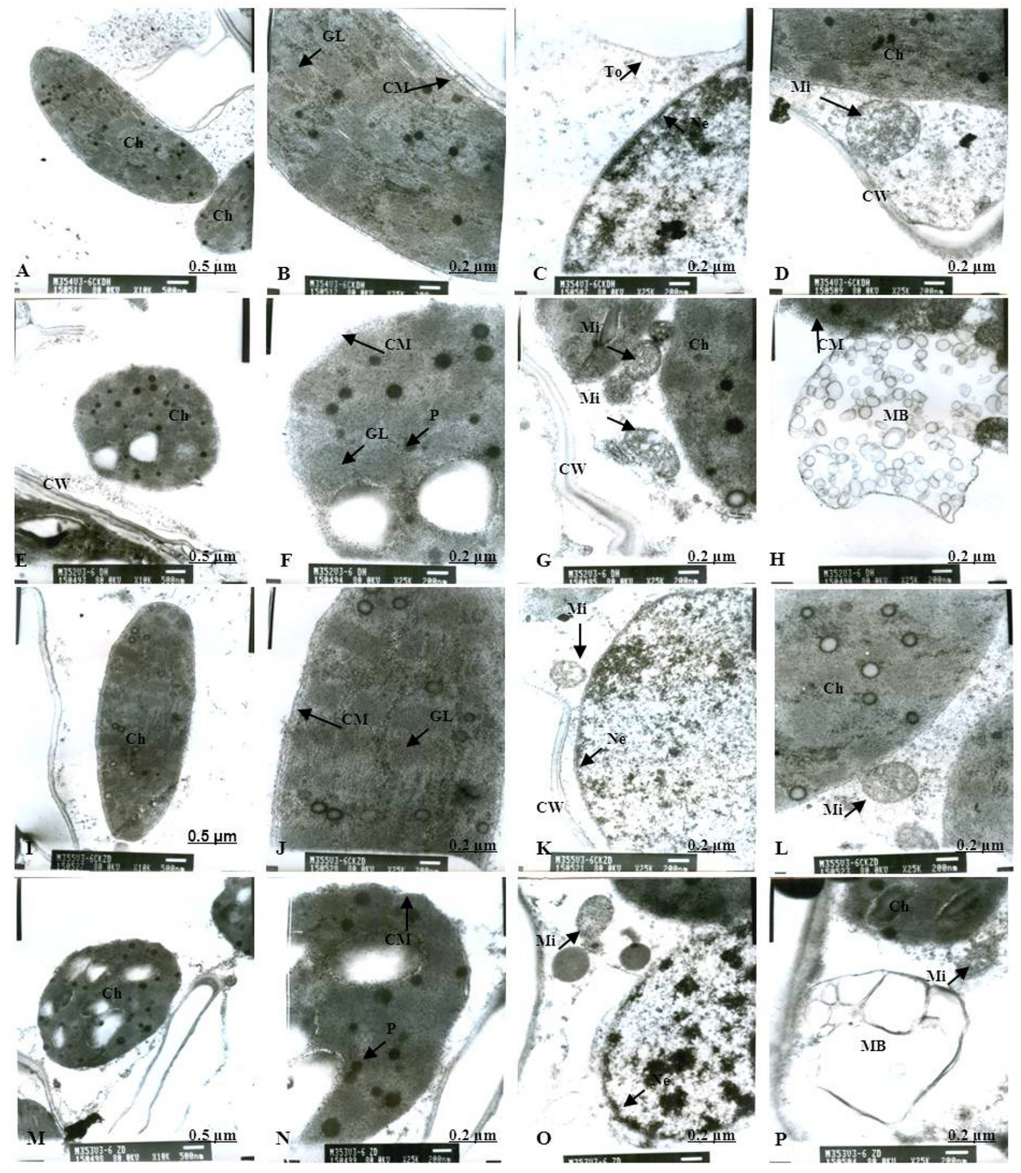
Effects of waterlogging at V3 for 6 d on the mesophyll cell and chloroplast ultrastructure. (A) represents the complete picture of mesophyll cells and topography (×10K) of mesophyll cells in chloroplast of DH605 in CK; (B), (C) and (D) represents ultrastructure (×25K) of mesophyll cells of DH605 in CK; (E) represents the complete picture of mesophyll cells and topography (×10K) of mesophyll cells in chloroplast of DH605 in V3-6; (F), (G) and (H) represents ultrastructure (×25K) of mesophyll cells of DH605 in V3-6; (I) represents the complete picture of mesophyll cells and topography (×10K) of mesophyll cells in chloroplast of ZD958 in CK; (J), (K) and (L) represents ultrastructure (×25K) of mesophyll cells of ZD958 in CK; (M) represents the complete picture of mesophyll cells and topography (×10K) of mesophyll cells in chloroplast of ZD958 in V3-6; (N), (O) and (P) represents ultrastructure (×25K) of mesophyll cells of ZD958 in V3-6; Ch: chloroplast; CM: chloroplast membrane; GL: grana lamella; Mi: mitochondria; Ne: nuclear envelope; P: particles; CW: cell wall. To: tonoplast; MB: multivesicular body.

Normal chloroplast configuration and structure in mesophyll cells are basic for ensuring normal leaf photosynthesis of maize. In this study, chloroplast configuration became irregular, and chloroplast number in mesophyll cells was reduced accordingly after waterlogging. The number of chloroplasts in mesophyll cells were decreased by 20.93% and 11.59% for DH605 and ZD958, respectively, after waterlogging for 3 d at V3, and decreased by 32.50% and 40.63% after waterlogging for 6 d compared to that of CK, respectively. After waterlogging for 3 d at V6, chloroplast number in mesophyll cells were decreased by 8.82% and 16.89% for DH605 and ZD958, and decreased by 23.13% and 25.23% after waterlogging for 6 d compared to that of CK, respectively. After waterlogging for 3 d at 10VT, the comparison of waterlogging treatments with CK showed no significant differences in the number of chloroplasts. Chloroplast number was decreased by 5.11% and 6.32% for DH605 and ZD958, respectively, after waterlogging for 6 d at 10VT compared to that of CK (Tables 4 and 5). In addition, edema was evident in parts of chloroplasts that were shorter and wider than other chloroplasts. The external of chloroplasts changed from long and oval to elliptical or almost circular (Figs 5E and 5M, 6E and 6M). Chloroplast morphology was most susceptible to damage when waterlogging occurred at V3, followed by V6 and 10VT. The length and width of chloroplasts were reduced significantly with increasing waterlogging duration, with the most significant reduction in V3-6 with decreases in length and width of 17.13% and 27.43%, respectively, for DH605, compared with CK, while the corresponding values for ZD958 decreased by 27.43% and 42.92% respectively (Tables 4 and 5). In addition, chloroplast number was significantly positively related to chl (a+b), *P*_n_, and grain yield (Table 6).

**Table 4:** Effect of waterlogging on chloroplast ultrastructure characteristics in mesophyll cells of DH605.

Waterlogging stage	Waterlogging duration	Treatment	Chloroplast number per mesophyll cell	Grana number per chloroplast	Lamellae number per grana	Chloroplast size	
						Length	Width
						(μm)	(μm)
**V3**	3	T	5.1b	16.5b	5.1b	12.7b	9.1a
		CK	6.5a	20.7a	9.8a	15.3a	6.9b
		±CK%	-21.5	-20.3	-48	-17	31.9
	6	T	5.4b	16.5b	7.4b	13.6b	11.1a
		CK	8.0a	22.6a	16.8a	16.5a	6.4b
		±CK%	-32.5	-27	-56	-17.6	73.4
**V6**	3	T	6.2b	22.2b	11.1b	14.4b	8.8a
		CK	6.8a	27.0a	15.3a	16.4a	6.7b
		±CK%	-8.8	-17.8	-27.5	-12.2	31.3
	6	T	6.2b	19.2b	10.9b	14.9b	9.4a
		CK	8.0a	25.7a	16.9a	17.2a	6.8b
		±CK%	-22.5	-25.3	-35.5	-13.4	38.2
**10VT**	3	T	8.1a	21.5b	21.2b	15.2a	8.4a
		CK	8.4a	23.1a	26.4a	16.2a	7.0b
		±CK%	-3.6	-6.9	-19.7	-6.2	20
	6	T	8.4a	21.3b	20.1b	15.1b	8.2a
		CK	8.8a	23.8a	26.4a	16.9a	6.4b
		±CK%	-4.5	-10.5	-23.9	-10.7	28.1
**ANOVA**							
**Waterlogging period (P)**			**	*	*	*	**
**Waterlogging duration (D)**			*	NS	*	NS	NS
**P×D**			*	*	*	NS	*

T: waterlogging; CK: no waterlogging; ±CK% = (CK value-T value)/CK value×100.

Values fallowed by a different small letter within a column are significantly different (P = 0.05) according to L. S. D. (t).

NS: Not significant.

* Significant at the 0.05 probability level

** Significant at the 0.01 probability level

**Table 5:** Effect of waterlogging on chloroplast ultrastructure characteristics in mesophyll cells of ZD958.

Waterlogging stage	Waterlogging duration	Treatment	Chloroplast number per mesophyll cell	Grana number per chloroplast	Lamellae number per grana	Chloroplast size	
						Length	Width
						(μm)	(μm)
**V3**	3	T	7.3b	18.2b	8.3b	13.2b	9.4a
		CK	8.2a	24.7a	10.7a	16.6a	6.7b
		±CK%	-11	-26.3	-22.4	-20.5	40.3
	6	T	4.8b	17.2b	9.8b	12.3b	8.6a
		CK	8.0a	24.6a	14.4a	17.0a	6.0b
		±CK%	-40	-30.1	-31.9	-27.6	43.3
**V6**	3	T	6.2b	19.4b	12.7b	14.3b	8.5a
		CK	7.4a	24.9a	16.3a	17.3a	6.8b
		±CK%	-16.2	-22.1	-22.1	-17.3	25
	6	T	6.1b	19.1b	10.5b	13.2b	9.5a
		CK	8.2a	25.8a	16.2a	16.4a	6.7b
		±CK%	-25.6	-26	-35.2	-19.5	41.8
**10VT**	3	T	8.1a	22.9a	23.1b	16.0a	8.1a
		CK	8.6a	23.8a	25.8a	16.9a	7.3a
		±CK%	-5.8	-3.8	-10.5	-5.3	11
	6	T	8.2a	22.4b	21.0b	15.1b	8.8a
		CK	8.7a	23.8a	25.9a	16.8a	7.5b
		±CK%	-5.7	-5.9	-18.9	-10.1	17.3
**ANOVA**							
**Waterlogging period (P)**			**	*	*	*	**
**Waterlogging duration (D)**			*	NS	*	NS	NS
**P×D**			*	*	*	NS	*

T: waterlogging; CK: no waterlogging; ±CK% = (CK value-T value)/CK value×100.

Values fallowed by a different small letter within a column are significantly different (P = 0.05) according to L. S. D. (t).

NS: Not significant.

* Significant at the 0.05 probability level

** Significant at the 0.01 probability level

**Table 6:** Correlation analyses among chloroplast ultrastructure, photosynthetic characteristics, and yield.

Correlation coefficient	Chloroplast number per mesophyll cell	Grana number per chloroplast	Lamellae number per grana	Chl (a+b)	Pn	Yield
**Chloroplast number per mesophyll cell**	1					
**Grana number per chloroplast**	0.724**	1				
**Lamellae number per grana**	0.821**	0.573**	1			
**Chl (a+b)**	0.665**	0.460*	0.790**	1		
**Pn**	0.843**	0.680**	0.805**	0.729**	1	
**Yield**	0.745**	0.850**	0.572**	0.595**	0.817**	1

** Correlation is significant at the 0.01 level

* Correlation is significant at the 0.05 level

### Chloroplast ultrastructure

**Fig 7:**
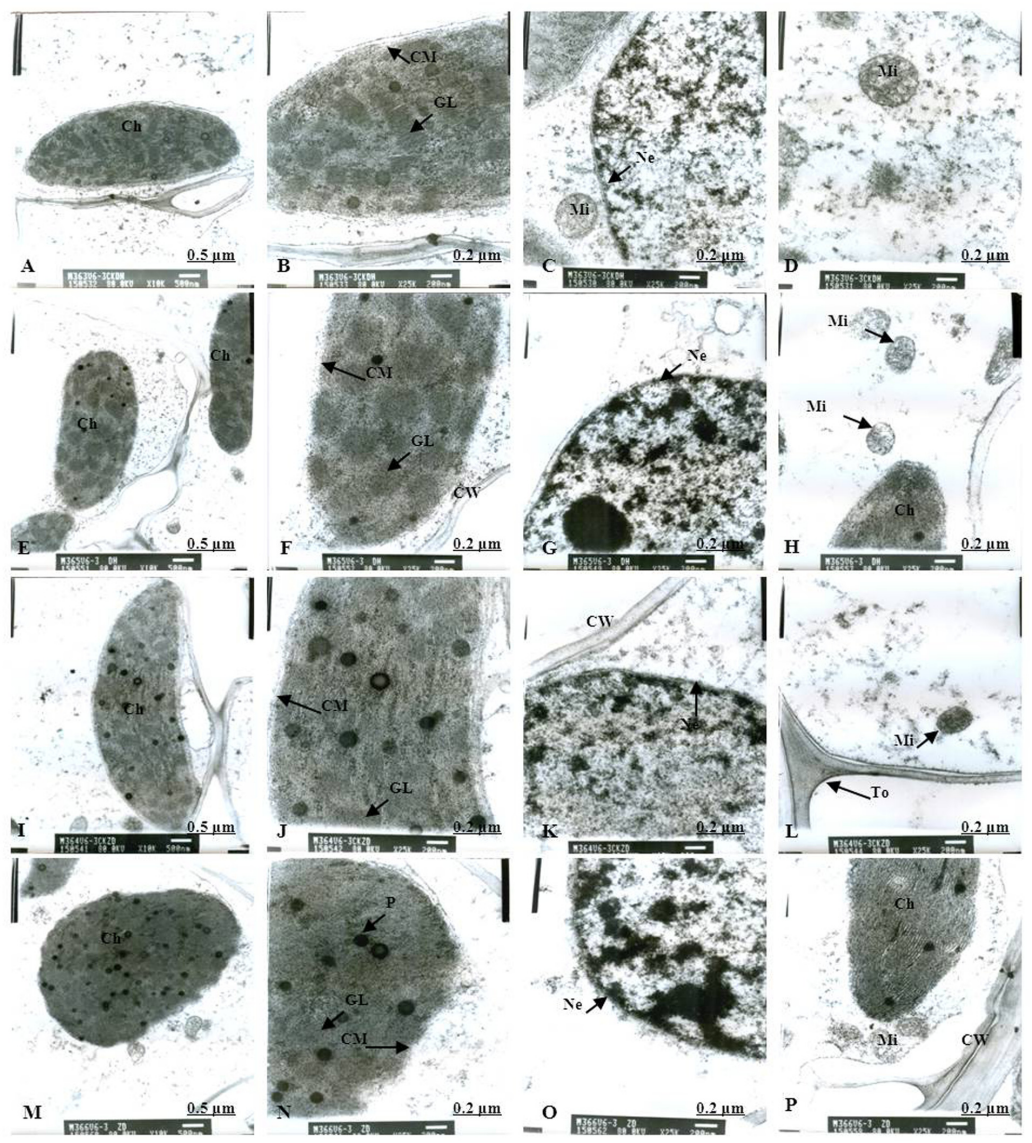
Effects of waterlogging at V6 for 3 d on the mesophyll cell and chloroplast ultrastructure. (A) represents the complete picture of mesophyll cells and topography (×10K) of mesophyll cells in chloroplast of DH605 in CK; (B), (C) and (D) represents ultrastructure (×25K) of mesophyll cells of DH605 in CK; (E) represents the complete picture of mesophyll cells and topography (×10K) of mesophyll cells in chloroplast of DH605 in V6-3; (F), (G) and (H) represents ultrastructure (×25K) of mesophyll cells of DH605 in V6-3; (I) represents the complete picture of mesophyll cells and topography (×10K) of mesophyll cells in chloroplast of ZD958 in CK; (J), (K) and (L) represents ultrastructure (×25K) of mesophyll cells of ZD958 in CK; (M) represents the complete picture of mesophyll cells and topography (×10K) of mesophyll cells in chloroplast of ZD958 in V6-3; (N), (O) and (P) represents ultrastructure (×25K) of mesophyll cells of ZD958 in V6-3; Ch: chloroplast; CM: chloroplast membrane; GL: grana lamella; Mi: mitochondria; Ne: nuclear envelope; P: particles; CW: cell wall. To: tonoplast; MB: multivesicular body.

**Fig 8:**
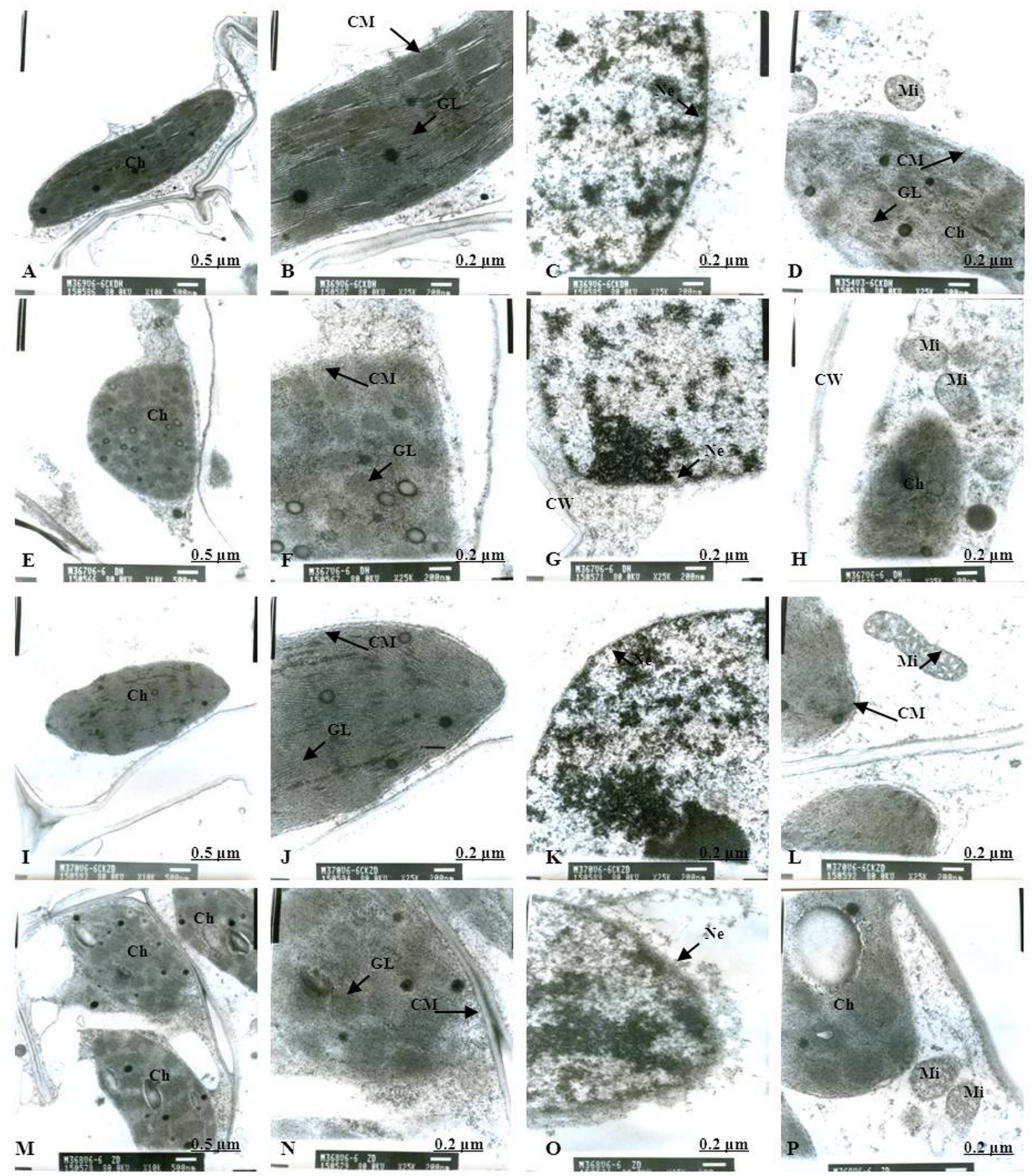
Effects of waterlogging at V6 for 6 d on the mesophyll cell and chloroplast ultrastructure. (A) represents the complete picture of mesophyll cells and topography (×10K) of mesophyll cells in chloroplast of DH605 in CK; (B), (C) and (D) represents ultrastructure (×25K) of mesophyll cells of DH605 in CK; (E) represents the complete picture of mesophyll cells and topography (×10K) of mesophyll cells in chloroplast of DH605 in V6-6; (F), (G) and (H) represents ultrastructure (×25K) of mesophyll cells of DH605 in V6-6; (I) represents the complete picture of mesophyll cells and topography (×10K) of mesophyll cells in chloroplast of ZD958 in CK; (J), (K) and (L) represents ultrastructure (×25K) of mesophyll cells of ZD958 in CK; (M) represents the complete picture of mesophyll cells and topography (×10K) of mesophyll cells in chloroplast of ZD958 in V6-6; (N), (O) and (P) represents ultrastructure (×25K) of mesophyll cells of ZD958 in V6-6; Ch: chloroplast; CM: chloroplast membrane; GL: grana lamella; Mi: mitochondria; Ne: nuclear envelope; P: particles; CW: cell wall. To: tonoplast; MB: multivesicular body.

**Fig 9:**
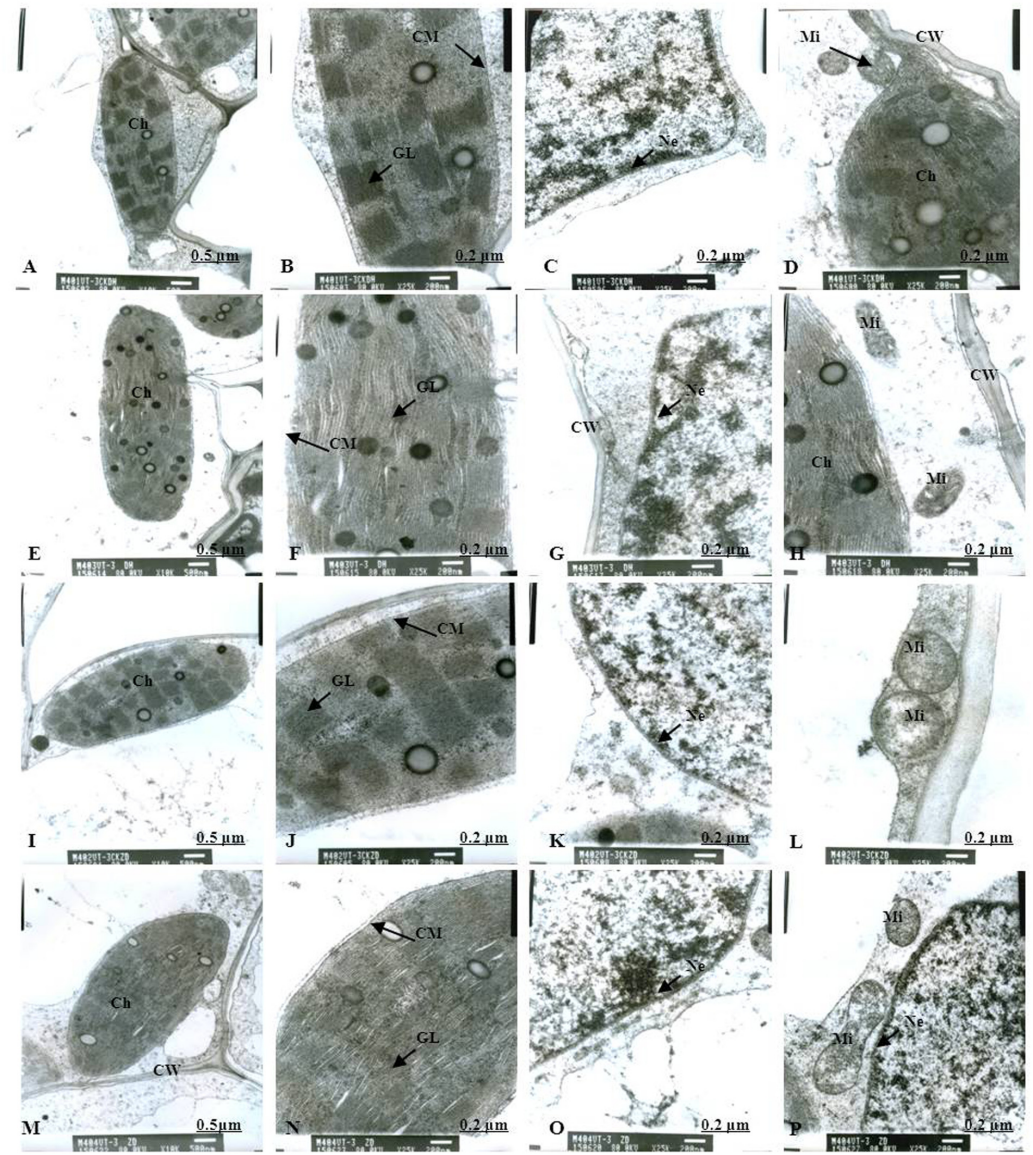
Effects of waterlogging at 10VT for 3 d on the mesophyll cell and chloroplast ultrastructure. (A) represents the complete picture of mesophyll cells and topography (×10K) of mesophyll cells in chloroplast of DH605 in CK; (B), (C) and (D) represents ultrastructure (×25K) of mesophyll cells of DH605 in CK; (E) represents the complete picture of mesophyll cells and topography (×10K) of mesophyll cells in chloroplast of DH605 in 10VT-3; (F), (G) and (H) represents ultrastructure (×25K) of mesophyll cells of DH605 in 10VT-3; (I) represents the complete picture of mesophyll cells and topography (×10K) of mesophyll cells in chloroplast of ZD958 in CK; (J), (K) and (L) represents ultrastructure (×25K) of mesophyll cells of ZD958 in CK; (M) represents the complete picture of mesophyll cells and topography (×10K) of mesophyll cells in chloroplast of ZD958 in 10VT-3; (N), (O) and (P) represents ultrastructure (×25K) of mesophyll cells of ZD958 in 10VT-3; Ch: chloroplast; CM: chloroplast membrane; GL: grana lamella; Mi: mitochondria; Ne: nuclear envelope; P: particles; CW: cell wall. To: tonoplast; MB: multivesicular body.

**Fig 10:**
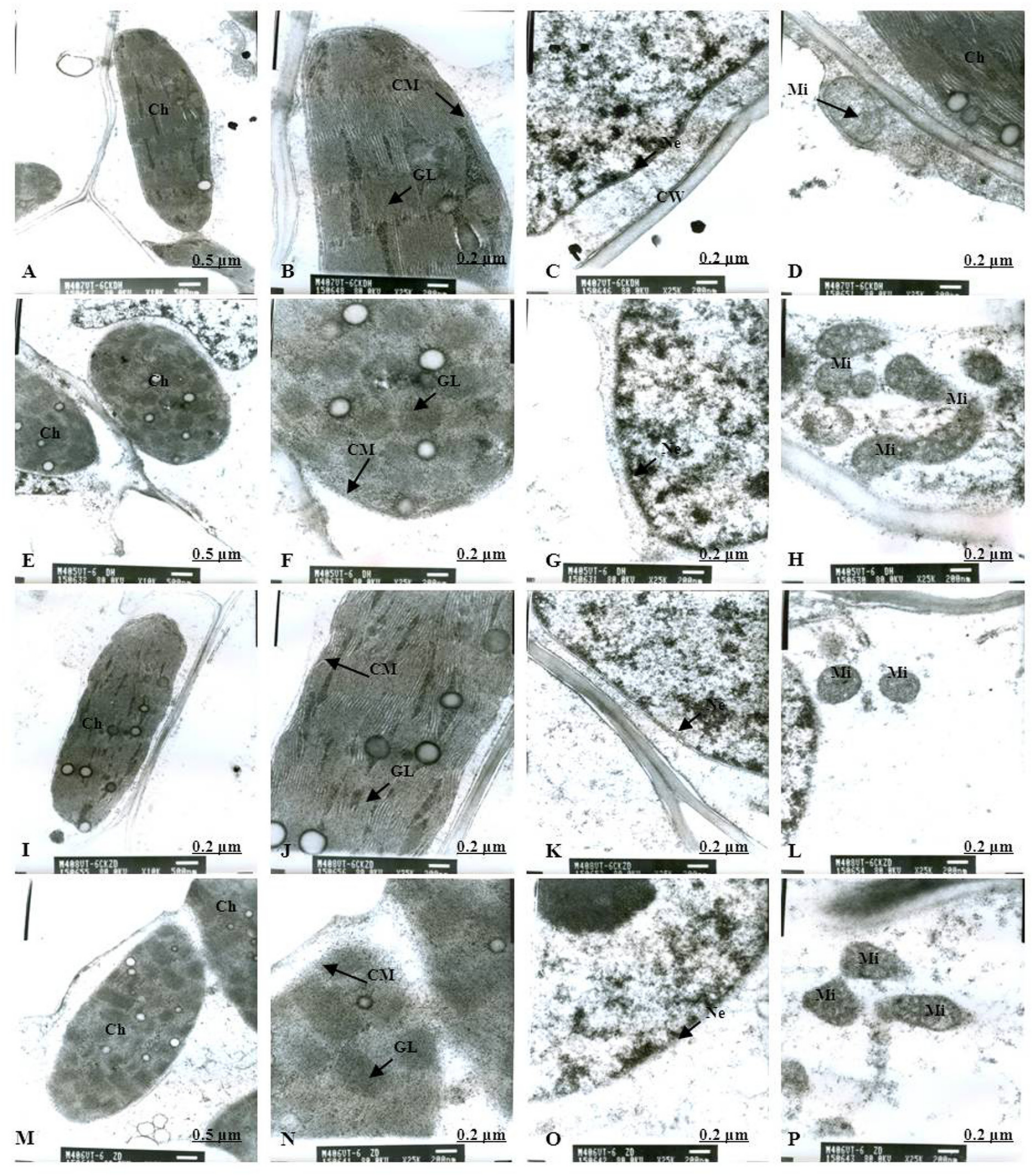
Effects of waterlogging at 10VT for 6 d on the mesophyll cell and chloroplast ultrastructure. (A) represents the complete picture of mesophyll cells and topography (×10K) of mesophyll cells in chloroplast of DH605 in CK; (B), (C) and (D) represents ultrastructure (×25K) of mesophyll cells of DH605 in CK; (E) represents the complete picture of mesophyll cells and topography (×10K) of mesophyll cells in chloroplast of DH605 in 10VT-6; (F), (G) and (H) represents ultrastructure (×25K) of mesophyll cells of DH605 in 10VT-6; (I) represents the complete picture of mesophyll cells and topography (×10K) of mesophyll cells in chloroplast of ZD958 in CK; (J), (K) and (L) represents ultrastructure (×25K) of mesophyll cells of ZD958 in CK; (M) represents the complete picture of mesophyll cells and topography (×10K) of mesophyll cells in chloroplast of ZD958 in 10VT-6; (N), (O) and (P) represents ultrastructure (×25K) of mesophyll cells of ZD958 in 10VT-6; Ch: chloroplast; CM: chloroplast membrane; GL: grana lamella; Mi: mitochondria; Ne: nuclear envelope; P: particles; CW: cell wall. To: tonoplast; MB: multivesicular body.

As for the CK plants, chloroplasts had a complete external envelope and clear boundary, and the thylakoid systems were well-developed (Figs 5A and 5I, 6A and 6I, 7A and 7I, 8A and 8I, 9A and 9I, 10A and 10I). The lamella structure pile folds were in order, and both grana lamella and stroma lamellae were arranged compactly and clearly (Figs 5B and 5J, 6B and 6J, 7B and 7J, 8B and 8J, 9B and 9J, 10B and 10J). By contrast, the chloroplast internal structure was deteriorated, and the numbers of grana and grana lamellae were reduced significantly to varying degrees after waterlogging. The negative effects varied with growth stage and waterlogging duration, with the most significant effects occurring at V3, followed by V6 and 10VT. The numbers of grana and grana lamellae were reduced significantly with increasing waterlogging duration, with the most significant reductions of 26.83% and 55.95% in V3-6 for DH605, respectively. Those of ZD958 decreased by 30.08% and 31.94% compared to those of CK, respectively (Tables 4 and 5). After waterlogging for 3 d at V3 and V6, chloroplasts were partially damaged, the external capsule grana lamellae were fuzzy and disordered, the interlayer gap became larger, and multi-vesicular bodies were occasionally found (Figs 5F and 5N, 7F and 7N). After waterlogging for 6 d at the same growth stage, most chloroplasts were similarly round and showed external envelope degradation (Figs 6E and 6M, 10E and 10M). In waterlogged treatments, the grana and substrate lamella were not clearly differentiated, the number of osmiophilic granule and multivesicular body was increased, and individual chloroplasts disintegrated (Figs 6F and 6N, 8F and 8N). After waterlogging for 3 d at 10VT, grana and grana lamellae were still well developed and exhibited only partial adventitia fractures. However, the lamellar structure was arranged loosely, and cracks among lamellae were evident. The grana lamellae gradually became twisted (Fig 9F and 9N). After waterlogging for 6 d at 10VT, chloroplasts became round, grana lamellae were blurred, and few osmiophilic granules were found (Fig 10F and 10N). In addition, the numbers of grana and grana lamellae were significantly positively related to chl (a+b), *P*_n_, and grain yield (Table 6).

### Mitochondrion structure

In normal leaf cells, mitochondria were in a random arrangement in the cytoplasm and were almost circular or elliptical. The double membrane structure was complete, and the crest was clearly visible (Figs 5D and 5L, 6D and 6L, 7D and 7L, 8D and 8L, 9D and 9L, 10D and 10L). However, waterlogging influenced mitochondrial structures to a certain extent. After exposure to waterlogging, mitochondria were in a clustered arrangement near chloroplasts, which increased the loss of energy produced by mitochondrial respiration when energy was in the transport process. Mitochondrial outer membrane dimmed, and fine-grained substance exosmosed and became diluted (Figs 5G and 5O, 6G and 6O, 7G and 7O, 8G and 8O, 9G and 9O, 10G and 10O). Mitochondrial longitudinal diameter became long and thread-like, and internal ridges lost their shape, dissolved gradually and lost their physiological function (Figs 6H and 6P, 10H and 10P). Thus, mitochondria were most susceptible to damage when waterlogging occurred at V3, followed by V6 and 10VT, and damage increased with increasing waterlogging duration.

### Membrane structure

The entire membrane structure plays an important role in the normal physiological functioning of cells. Under waterlogging stress, the cytoderm structure became incomplete and exhibited indistinct gradation, lower density and a loose edge. After waterlogging for 3 d at V3 or V6, only the edges of nuclear, cell, vacuole and chloroplast membranes were destroyed. Plasmolysis and cell membrane degradation were observed occasionally (Figs 5F, 5H, 5N and 5O, 7F, 7N and 7P). By contrast, after waterlogging for 6 d, complete and defined membrane structures were rarely found in either mitochondria or chloroplasts. Most membranes of cells, vacuoles and chloroplasts were dissolved (Figs 6F and 6N, 10F and 10N), and plasmolysis and cell membrane degradation were more serious (Figs 6G and 8G). Furthermore, some membranes were degraded and liquefied into multivesicular body (Fig 6H and 6P). However, the effects of waterlogging at 10VT on membrane structures were not significant (Figs 9 and 10).

## Discussion

Waterlogging significantly decreased leaf area and LAI of summer maize. Previous studies showed that waterlogging significantly reduced LAI and net photosynthetic rate ([9, 19] and [20]), although Vandoorne *et al*. (2014) found that waterlogging could also increase LAI for some plants in specific anoxic conditions [21]. Our study showed that significant reductions in LAI occurred in response to waterlogging. Furthermore, waterlogging caused a reduction in leaf area, accelerated the senescence process, thereby resulting in negative photosynthetic properties. Our results were also in agreement with the previous study that waterlogging reduced chlorophyll content, resulting in a decline in crop leaf photosynthesis [20, 22], which indicated that waterlogging affected leaf photosynthesis of summer maize, and weakened the photosynthetic assimilation capacity.

The negative effects of waterlogging on LAI and chlorophyll content resulted in a decline in leaf photosynthesis. In our study, waterlogging reduced *P*_n_ accompanied by pronounced reductions of *G*_s_, *T*_r_, and *C*_i_, illustrating that reductions in photosynthesis after waterlogging were mainly due to stomatal factors. Chlorophyll fluorescence is an efficient tool for indicating changes in functions of photosynthetic apparatus, which can be damaged by waterlogging [23, 24]. However, the declines in *F*_v_/*F*_m_ and *Φ*_PSII_ were observed after waterlogging, indicating waterlogging damage to PSII of summer maize. Thus, the photosynthesis potential energy of PSII was reduced, leading to declines in photosynthetic rate and photosynthetic characteristics [7], and ultimately resulted in a significant reduction in grain yield after waterlogging.

The reductions of chlorophyll content and photosynthetic capacity were mainly correlated with the disturbance of chloroplast morphology and ultrastructure of functional leaves [10, 11]. Chloroplasts and mitochondria are major ROS-generating sites under stress conditions [25]. It has been observed that the chloroplast membrane ruptures under chilling injury [12] and heat stress [25], and that thylakoid was disrupted under drought stress [11] and low light [12]. Our results also showed that chloroplast arrangement dispersed, the integrity of chloroplast ultrastructure was destroyed, their membranes and thylakoids were deliquescent, after waterlogging. As a result, the photosynthetic process was consequentially inhibited [26], and chlorophyll content and chlorophyll fluorescence parameters were reduced [25], eventually resulting in the degradation of leaf photosynthetic capacity. Moreover, some mitochondria became longer and dysfunctional gradually, their membranes were dissolved. These changes would inhibit leaf respiration, which was interdependent with photosynthesis [27]. Therefore, the effects of waterlogging on mitochondria further weaken the photosynthesis of summer maize.

The cell death of waterlogged plants might due to reduced stability of the membrane system structure, caused by the lack of ATP in the cell membrane [28, 29]. Our results showed that, membrane structure gradually damaged, which were similar with the results of damaged organelle membrane structures, as also reported by Pfister *et al*. [28] and Crawford *et al*. [29]. This result indicated that the effect of waterlogging on leaf cells was closely related to the destruction of membrane systems, causing apoptosis and the loss of photosynthetic capacity [30, 31]. In addition, our study showed that MDA content significantly increased after waterlogging at various stages indicating a negative impact of waterlogging on membrane integrity, and ultimately membrane deterioration. Such alterations would affect the ion exchange capacity of plasma membrane and some physiological activities linked to membrane functioning [32]. The damaged mesophyll cell ultrastructure of functional leaf induced by waterlogging would lead to a decline in leaf photosynthetic ability, resulting in grain yield reduction of summer maize.

## Conclusions

The reduction of photosynthesis induced by waterlogging, as a consequence of chloroplast, mitochondria and membrane alterations, resulting in a significant reduction in grain yield of summer maize. Summer maize was most susceptible to damage when waterlogging occurred at V3, followed by V6 and 10VT. Damage increased with increasing waterlogging duration.
